# Analysis of Cement Deterioration in Outdoor High-Voltage Insulator

**DOI:** 10.3390/ma12244201

**Published:** 2019-12-13

**Authors:** Taeyong Kim, Simpy Sanyal, Ja-Bin Koo, Ju-Am Son, In-Hyuk Choi, Junsin Yi

**Affiliations:** 1 College of Information and Communication Engineering, Sungkyunkwan University, Suwon, Gyeonggi-Do 16419, Korea; absolutely@skku.edu (T.K.); Sanyalsimpy@gmail.com (S.S.); 2 KEPCO Research Institute, Daejeon 34056, Korea; kilik321@kepco.co.kr (J.-B.K.); sonjuam@kepco.co.kr (J.-A.S.)

**Keywords:** porcelain insulators, cement, OHTL, alkali silica reaction, fuchsine, indicator, hydrogen concentration

## Abstract

Suspension type porcelain insulators used in overhead transmission lines comprise metal, ceramic, and cement. The deterioration of cement can lead to mechanical separation. For the degradation analysis, varied sizes of pores ranging from a few µm (capillary pores) to tens to hundreds of mm (detectable by naked eyes) were considered. Cracks that were hard to view with naked eyes were identified by staining with a fuchsine solution. The hydrogen ion concentration and pH value indicate the extent to which the cement is deteriorated. The longer the cement is used, the lower its pH value. High mechanical strength is considered an important advantage of porcelain insulators, and it may decline, if the cement is used for a longer period of time. Water ingress may also occur, resulting in expansion, due to the rehydration of the cement. The process and mechanism of expansion of cement, due to infiltration of water were described. As a method of analysis, a universal indicator was employed to evaluate the pH changes in cement. It was observed that the pH value was 12–13 for new products. However, for products that were used for 52 years, the pH value was under 7, which indicated an acidic tendency, due to deterioration.

## 1. Introduction

**Figure 1:**
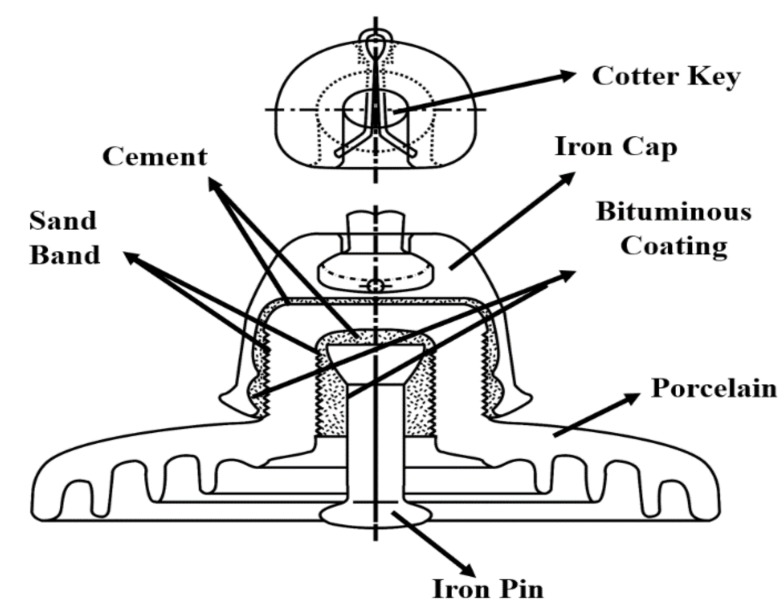
Cross section of suspension type porcelain insulator used for 154 kV transmission line.

Suspension type insulators used in overhead transmission lines (OHTLs) were first designed in 1942 and are still in use. Since its initial production, it has been strictly managed with an international standard and has been generalized around American National Standards Instituteand International Electro-technical Commission. The electrical and mechanical performance should fulfill the generalized specifications. Some detailed specification may differ depending on the countries [1,2]. Manufacturers produce their own insulators with better characteristics, while still meeting the standards. The cross-sectional view of the porcelain insulator, which is the subject of this study, is shown in Figure 1. It is a porcelain insulator that satisfies 25,000 lb of suspension type used in a 154-kV-transmission line. Insulation characteristics are very important for smooth power supply through the power transmission lines. The design and material of the insulators vary according to the processing method. These insulators are produced worldwide by contributing a considerable amount of effort to ensure that their product is in line with international standards [3,4].

Once installed, there are difficulties encountered in conducting the maintenance without disturbing the power supply to the general living environment. Supply stability and continuity are essential, and replacing the insulators is difficult. Currently, in most countries, replacements are provided, when major damages can be visibly noticed. It is very difficult to predict the lifetime of insulators. Many researchers state that an insulator can be used for approximately 30–60 years [5].

Several insulation devices demonstrate a diverse lifespan and are externally installed. Therefore, it is not possible to avoid exposure to pollutants. Depending on the usage environment, these devices may undergo deterioration, due to various factors relating to the city, industrial area, and coast, and therefore, periodic replacements are essential. Power outages caused by problems with insulators and ground faults, as well as short circuits caused by falling insulators can entail significant economic loss and additional human and material damage [6,7]. Predicting how long the insulator can be used without causing any problems is important. In terms of electrical characteristics, no arc discharge should be encountered.

The insulator must withstand instantaneous overvoltage from the environment. Even if the insulation characteristic declines, due to humidity, it should not decline to the extent of causing a leakage. In terms of mechanical properties, a reasonable amount of mechanical strength is needed to endure the weight of the conductors between transmission lines under varied weather conditions, such as ice, snow, rain, wind, and earthquakes [8,9]. Diagnosing the lifespan of the insulator devices, during when their usage is reliable, is of significant advantage. There are diverse deterioration factors, and it is not known as to how these factors affect insulator devices. A porcelain insulator includes cement, iron, and ceramics, which are three different structures, with a bitumen coating and sand band between them. As such structures are composite, diagnosing the lifespan of insulators is complicated and difficult to define. Therefore, there are challenges associated withidentifying the most appropriate time to replace the insulators in a transmission line, before accidents occur [10,11].

**Figure 2:**
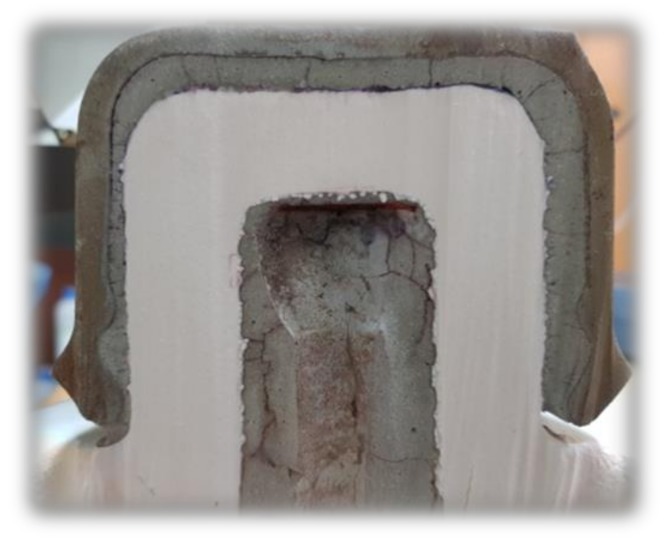
Cement paste surrounding cap of porcelain insulator.

In this study, we propose a method for diagnosing the degradation of cement paste in insulators and a method for diagnosing the lifetime of cement as depicted in Figure 2 [12]. An intensive analysis focusing on the cement life and not on the life of all parts of the insulator is performed. The method of diagnosing the life of cement includes measuring the pH variations by employing the universal indicator. The deterioration behavior of cement in the porcelain insulators is estimated using a universal indicator to observe the pH variation with respect to age [12,13,14]. The initial pH of the cement is 12–13 indicating alkaline characteristics. As degradation progresses, the hydrogen ion concentration decreases from neutral to acidic. In building structures, it is a common practice to proceed with reconstruction, when pH declines below neutral [15,16].

Alternatively, three-dimensional computed tomography (3D-CT) can be used to predict the deterioration of cement through internal imaging without damaging the insulation. The deterioration of cement is detected, and the location of the generation of pores is confirmed to be around the gap between the pin and porcelain. Moisture penetration into this gap causes cracks and pores to be enlarged, due to the freeze-thawing action that happens in accordance with the external environment. There is also the possibility of radial cracking of porcelain from cement expansion. When rough surfaces are caused, due to the deterioration of porcelain insulator, the generated charges are concentrated in the cement resulting in the possibility of corona discharging [17,18]. Pores are generated in the cement of the cap, due to the concentration of the electric field. The degradation of cement occurs in locations, where electrical stress is applied.

The process of hydration of cement and subsequent procedures are explained as follows. Clinker, which is the main component of cement used in porcelain insulators, comprises Alite, Belite, Aluminate, and Ferrite [19,20]. Alite and Belite hydrate to produce C-S-H gels and Ca(OH)_2_ [21]. Low crystalline calcium silicate hydrate is known as C-S-H bonding, and its chemical composition and crystallinity depend on production conditions. Immediately after the hydration reaction, pH rises rapidly, due to H_2_O invasion and the separation of Ca ions, resulting in the development of a low-density CS layer on the surface and causing some crystal growth [22,23,24]. The aluminate and ferrite crystal phases react with water, stabilize with C_4_AH_19_ and C_2_AH_8_ hydrates under rapid hydration, and then hydrate with stable C_3_AH_6_ over time [25]. As the hydration reaction is very rapid, it is difficult to cure, when only cement is being used. Therefore, gypsum may be added to produce ettringite hydrate, which is hydrated using C_3_A hydrate [26,27,28].

The degree of expansion or contraction that results during the process of hydrate formation or deterioration with age can be classified according to the type of cement. Most of the types of cement undergo an expansion and contraction process before hardening; however, during hardening and rehydration, cracks may occur, due to continuous expansion resulting from moisture supply [29,30]. The types of pores generated during the process of cement expansion can be divided based on their sizes [31,32]. Small µm cracks, which are not visible to the naked eye, are stained with the fuchsine solution so that they are visible during the determination of the extent of cement deterioration [33,34].

## 2. Experiments

To analyze the porcelain insulator, a nondestructive analysis was performed with 3D-CT analysis equipment using X-ray, which is a representative non-destructive analysis. Samples were cut with a waterjet, and a fracture analysis was performed using scanning electron microscopy (SEM), energy-dispersive X-ray spectroscopy, and fuchsine solutions.

An Aqua waterjet or a pure waterjet was employed for the purpose of waterjet processing. This is a technology that compresses pure water to ultra-high pressure over 200–400 MPa using a high-pressure pump and then sprays it on the surface of the material at two to three times the speed of sound using a nozzle, such as a sapphire or a diamond [35]. The waterjet was employed to avoid thermal damage to insulator samples. The processing size of the sample was analyzed in terms of cubes of height 5–10 mm so that it can be measured in conventional equipment. With the 3D-CT analysis, the 2D data obtained after rotating transmission scanning using X-ray was reconstructed into digital data. It can also be imaged in 3D. However, the X-rays may be attenuated and difficult to measure because of the complex structure of the porcelain insulator (e.g., thick metal cap). Another reason is that they have to penetrate three layers in the sequence of metal, cement, and ceramics. The voltage of the X-ray tube is 240 kV/450 kV, and the tube power is 320 W/400 W. The output power varies depending on the voltage. As the porcelain insulator is a combination of metal, cement, and ceramic, the decomposition ability is excellent, only when the tube with a high output voltage is used. A 400 mm × 400 mm detector was used in our experiment. The more expensive the equipment, the larger the detector size and the better the resolution.

The defect size and distribution tendency could be analyzed by visualizing information related to size measurement and position distribution of the internal pores, cracks, and foreign material in the region of interest. The specific spots of cracks can be analyzed using SEM. However, it is very difficult to locate and analyze the part, where alkali–silica reaction (ASR) has occurred Hitachi &Horiba/S4800&EDS opera having resolution 1.0 nm at 15kV. Lower magnification of the instrument is 20~2000×, whereas higher magnification is 100~800,000×. Hitachi is a multinational company based on Tokyo city of Japan.

With fracture analysis, it is possible to visually observe the level of cracks caused due to deterioration by dyeing cracks that were difficult to view with naked eyes using the fuchsine solution. If it is used as an undiluted solution, all parts may look dyed. Therefore, it is recommended to dilute this solution to 5% with alcohol or water. The change of the hydrogen ion concentration of cement in the insulator component was analyzed using the universal reagent test. Many experiments are conducted on building structures to determine the reconstruction. The pH of the initial cement or concrete is 12 or higher. The hydrogen ion concentration tends to decrease, due to water penetration and deterioration with age. When the pH declines under 10, it indicates that the corrosion of the metal inside is very high. If it declines under 7, it indicates that the mechanical capacity is highly reduced, and the insulator may be ineffective. This could be due to the reduction of the compressive stress. No specific applications have been conducted in the field of insulators; however, experiments conducted on building structures can provide some insight, because the internal structure is not much different across various buildings. As an experimental method, the indicator was dyed on a thin paper, and this paper was attached to its cutting edge. However, because of the moisture in the paper, the change of color, due to the concentration of hydrogen ions was difficult to classify. Applying the indicator to the cut insulator cross-section using a spray with a thin nozzle enabled the indicator particles to change colorimmediately. The change of color could then be easily classified. The SEM analysis focused on the crack sites after cutting the samples to similar sizes and applying PT coating. SEM is more suitable for measurement purposes in the case of the cement parts than FESEM (field emission SEM), as FESEM requires a high vacuum column. A trace of gas flows from the cement can break the high vacuum column.

## 3. Results and Discussion

**Figure 3:**
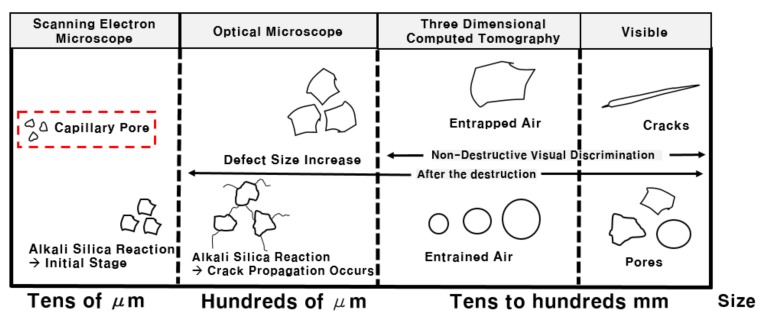
Classification table according to the size of pores in cement [17].

**Figure 4:**
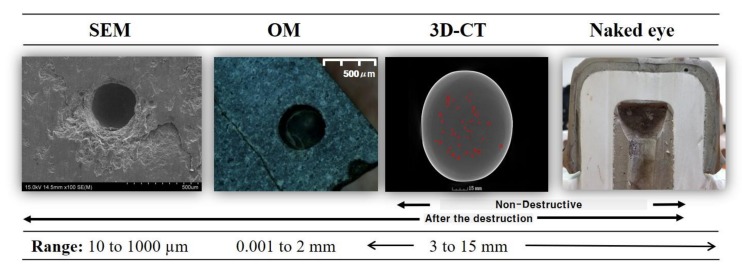
Pore size analysis according to the size of pores in cement.

Portland cement is used for constructing the 154-kV-porcelain insulators. Previous works it has been investigated that the pores in the cement vary in size from a few µm to mm, and they can be classified, as shown in Figure 3 It has also been reported that it exists in various sizes, ranging from the size that can be measured and observed with SEM to the size that can be visible to the naked eye as shown in Figure 4 [17]. The smallest pores can cause a capillary phenomenon. Due to the soil moisture tension, there are several micro-sized capillary pores that can contain moisture, and water penetrates into these pores, which is the starting point of microcracks that occur, due to the alkali–silica reaction (ASR) [35]. Larger than the capillary pores are the pores of several hundred µm that can be measured with an optical microscope. The ASR causes the capillary pores to be cracked [36]. As the cracks and voids grow larger, the cracks grow in the size of tens to hundreds of millimeters, which can be measured using 3D-CT without damaging the sample as performed by authors [14]. However, as the porcelain insulator is a composite material of metal, cement, and ceramic, it is not easy to measure the cracks because of X-ray scattering. Thus, it has been advised to perform 3D-CT with a very high output power for the analysis [17]. Efforts are taken to produce as little pores as possible using air-entraining water-reducing agents. Some of these pores grow in size to a few millimeters, which are similar to entrapped air.

Finally, if pores or cracks can be observed with the naked eye, the insulation or mechanical ability of the insulator is considered very low [37]. In general, cement is cured from a liquid to a solid, and when it is dried, the moisture in the capillary pores inside the cement evaporates, and the surface tension of the capillary pores and waterways increases, causing shrinkage [38]. As humidity increases, the capillary absorbs water and expands, when surface tension decreases [39]. Cement shrinks, as it hardens after fabrication; however, the most important factor is that dry shrinkage, due to evaporation of water causes cracks in the cured product.

**Figure 5:**
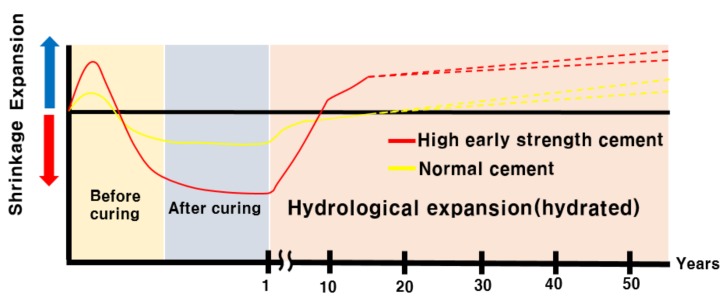
Contraction behavior, due to the service life of cement inside the porcelain insulator.

Figure 5 shows the shrinkage and expansion of cement inside the insulator with age with respect to normal and high-early-strength-cements. Manufacturers of porcelain insulators generally use the normal cement or high-early-strength cement, while may also use other types of cement. Less than one-year-old cement is not hardened completely. When the insulator is serviced after initial hardening, the cement may be rehydrated, due to moisture infiltration.When the compressive strength is elevated, and the cement is hardened by curing, moisture penetrates into the pores of porcelain and expands, because there is no coating film to prevent the penetration of moisture. It is difficult to define the number of years of use, after which that the crack may occur, due to expansion, because it depends on the service environment, leakage current if any, and the movement of ions. Swelling and cracking do not happen within a short period of time. The pH value declines, as the hydrogen ion concentration increases.

**Figure 6:**
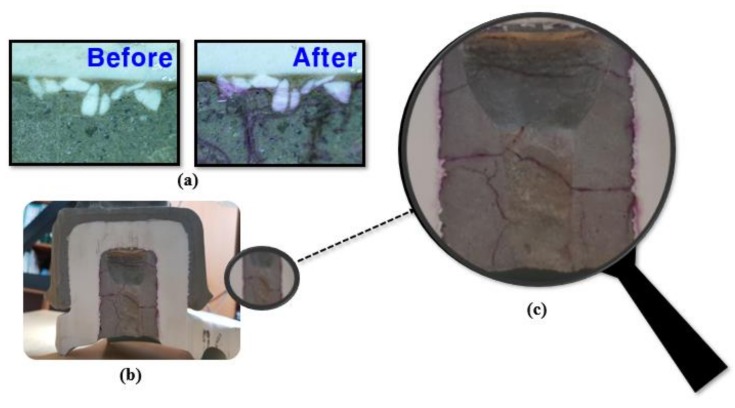
Crack detection using Fuchisne solution. (**a**) Before and after using fuchsine solution, (**b**) cross section after using solution, (**c**) enlarged cross section after using solution for visual inspection.

This can lead to swelling, which can eventually result in cracking and deterioration of mechanical properties. Degradation of mechanical properties, crevice, and swelling lead to cracks in the cement parts inside the insulator [40,41]. The results of using the fuchsine solution are shown in Figure 4. The solution is rosaniline hydrocholoride, magenta with the chemical formula of C_2_H_19_N_3_, HCl and soluble in water. It is mainly used to dye textiles or bacteria. If it becomes solid or thick, green crystals are formed. Therefore, it is diluted to 5% in water or ethanol [42]. Figure 6 shows the cracks before and after the use of the fuchsine solution. It can be observed that the cracks that were not visible to the naked eye before are visible, after the solution is used. The size of the cracks visible to the naked eye after using the solution is mostly in the range of 300–400 µm. Cracks of lesser size demonstrate a width of approximately 100 µm. As mentioned earlier, the formation of cracks isclosely related to the pH values.

In building structures, rebuilding and maintenance decisions may be made based on the value of pH. The effect of pH caused on the inner pin of the porcelain insulator is summarized in Table 1. The pH of the cement is initially 12–13, which indicates that the cement is strongly alkaline; however, due to deterioration with age, the pH value declines, as the hydrogen ion concentration of the cement increases.

If pH is over 10, the corrosion of the internal metal is difficult. When pH is in the range of 7–10, the probability of corrosion of the iron pin is very high. The lower the pH, the more ions that can move. Therefore, a lower pH value can be dangerous [42].

**Figure 7:**
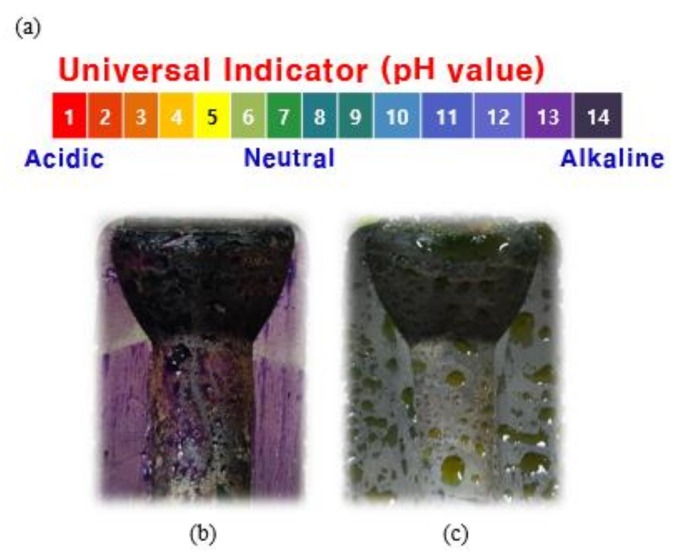
Color comparison of new and deteriorated products using the universal indicator on the insulator cement. (**a**) Color change by the pH value of the universal indicator, (**b**) purple color, due to the pH 12 of the new cement, (**c**) color change close to yellow, due to pH drop after 52 years use.

The change of pH in an insulator can be inspected using a universal indicator, and the color changes based on pH value, as shown in Figure 7a. This work considered two insulators, one new and other 52-year oldporcelain insulator. Both were cut in half using a waterjet and sprayed with a universal indicator on the internal cement part, as shown in Figure 7b,c. In the case of new insulator depicted in Figure 7b, after the hardening of the cement, the pH is observed to be approximately 13.

In the second case, where the porcelain insulator has been used for 52 years has shown a pH of approximately 7 as depicted in Figure 7c.

**Figure 8:**
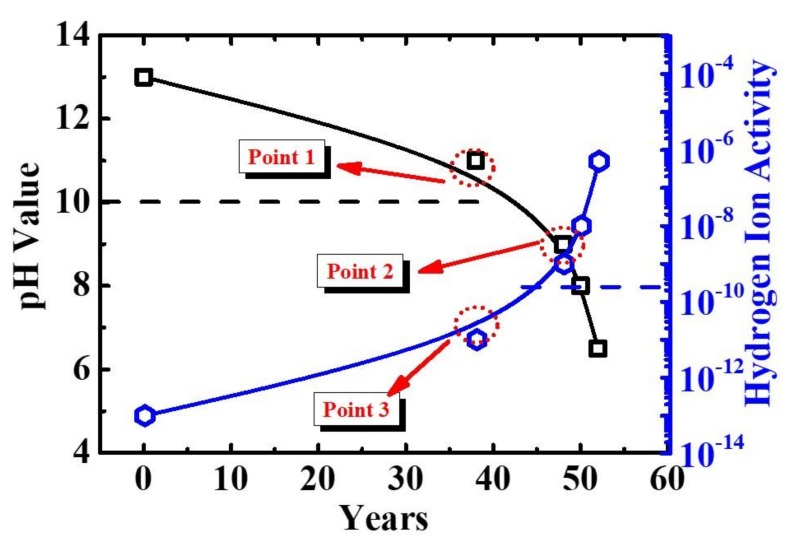
Changes in pH and H^+^ according to the used period in the cement of porcelain insulator.

This pH indicates the deterioration of cement, which implies that there can be defects in the mechanical properties of the insulator. When the pH value indicates strong alkalinity, a passivation film is formed on the surface of the iron pin so that it does not rust, even when oxygen is present. When the pH drops under 11, the pin may be rusted, due to the destruction of the passivation film. If iron is rusted, the volume may expand to more than 2.5 times. If the pH value is less than 7, it is considered to have been completely neutralized. It cannot be used on live lines because of the risks related to mechanical durability. The pH values can be expressed by the activities of H^+^ and OH^−^, as listed in Table 2. pH and H^+^ demonstrate a relationship of –log10 [H^+^], while H^+^ and OH^–^ demonstrate an inverse relationship. The change in pH and the amount of hydrogen ion activity, due to deterioration with age of the insulator are shown in Figure 8 When the pH drops under 9, the risk of mechanical properties increases at points 1 and 3, where the pH value is 10 and H^+^activity is 1 × 10^−10^.The age of the section is approximately 38 years, which is when corrosion is likely to occur on the pins inside the insulator. Point 2 is where pH and H^+^ activity intersect, pH drops under 9, and H^+^ activity is 1 × 10^−9^. It is the point, at which the alkalinity of cement drops sharply.

The age is approximately 42 years, which is when the deterioration of cement proceeds rapidly. At the age of 48 years, the mechanical strength of cement is exhausted, pH value is 7, and H^+^ activity is 1 × 10^−7^. The results of the study showed that the cement deterioration showed a decrease in pH, which is similar to that observed in the building. However, in the case of the insulator, the cement part is not coated. Therefore, moisture penetration into the capillary pores of the hardened cement is easier. Rehydration causes the cracks to expand and enlarge. As shown in Figure 8 the pH value decreases with age, and the cause of expansion is described in detail as follows:

Step 1:

Silica + Alkali → Alkali-Silica Gel (Sodium Silicate).(1)

Step 2:

SiO_2(Solid)_+ 2NaOH_(Solution)_ + H_2_O → Na_2_SiO_3_2H_2_O, (2)

SiO_2(Solid)_ + Na^+^_(aq.)_+(OH)^−^_(aq.)_→ NaHSiO_3(Solid)_, (3)

NaHSiO_3(Solid)_ + Na^+^_(aq.)_+(OH)^−^_(aq.)_→Na_2_SiO_3_xH_2_O. (4)

When the silica in the cement composition and strong alkalinity of the cement are combined as in Equation (1), the alkali-silica gel is formed, which is called sodium silicate. As mentioned in Equation (2), crystalline SiO_2_ and sodium silicate (2NaOH), combine and cause some cracks on the silica crystal. As shown in Equation (3), Na^+^ present in the cement react with basic OH^−^ to form NaHSiO_3_. When an insulator is used for a long time, these reactions happen in the mentioned sequence, and the essential conditions for the reaction are a high pH of the cement, moisture to penetrate, and reactive silica crystals. [43,44,45]. To prevent the occurrence of ASR, research has been reported that the use of low-alkali cement with an alkali content of less than 0.6% in cement can help [46].

**Figure 9:**
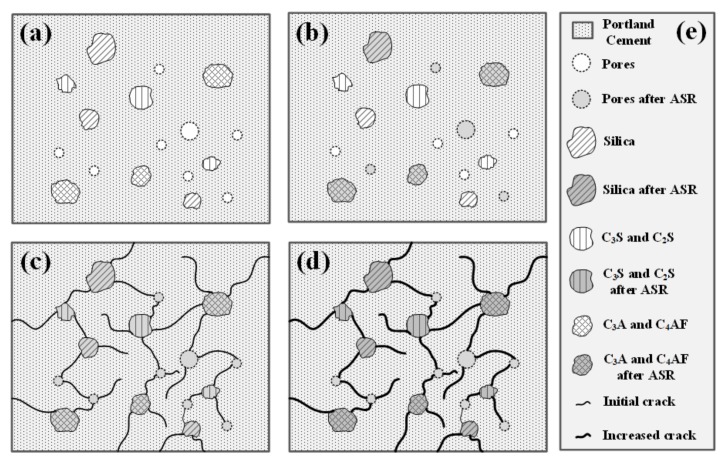
Diagram showing the alkali silica reaction mechanism of cement.

As pH decreases and internal corrosion starts, the processes related to cracking, expansion, and deterioration of cement happen, which are shown in Figure 9 Pores corresponding to the silica structure and capillary pores formed in Portland cement are demonstrated in Figure 9a As the cement is cured at a high temperature of 1260–1450 °C, minerals, such as crystal phase of tricalcium silicate (CaSiO_5_, 3CaO·SiO_2_) alite phase C3S, belite phase C_2_S crystal phase of dicalcium silicate (Ca_2_SiO_4_, 2CaO·SiO_2_), aluminate phase of tricalcium aluminate (Ca_3_Al_2_O_6_, 3CaO·Al_2_O_3_) containing trace silica, magnesium, sodium and potassium are produced. The ferrite phase C_4_AF crystal phase of tetra calcium alumino ferrite (Ca_4_Al_2_Fe_2_O_10_, 4CaO, Al_2_O_3_, Fe_2_O_3_), a chemically resistant compound contained in Portland with approximately 10% of C_3_A, is present.

Figure 9b shows that some water penetrated, and water is contained in pores corresponding to the capillary pores of cement. Four crystalline phases (C_3_S, C_2_S, C_3_A, and C_4_AF) react with water to cause cracks, which is shown in Figure 9c The deterioration proceeds, and the crystal phase undergoes ASR, due to moisture infiltration in accordance with steps 1 and 2 described earlier.

This is shown in Figure 9d. As a result, the insulator cement cannot guarantee the maintenance of mechanical properties, which is its primary role. The specific spots of cracks can be analyzed using SEM.However, it is very difficult to locate and analyze the part, where ASR has occurred [47,48].

**Figure 10:**
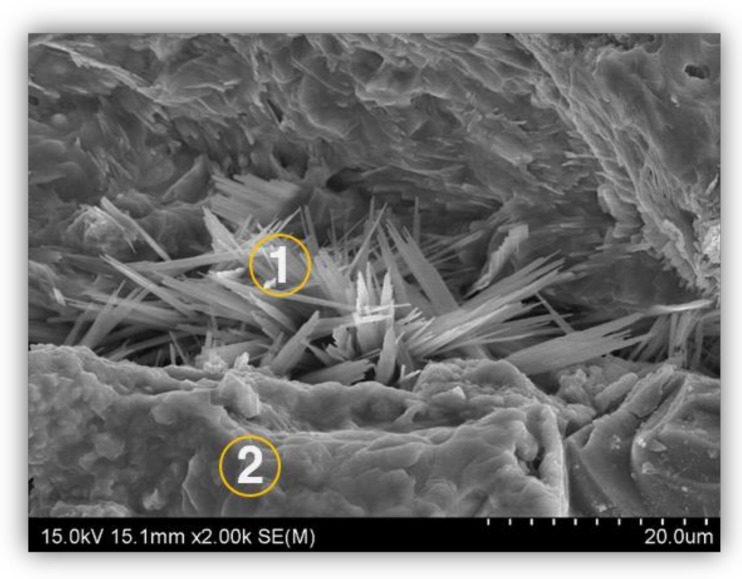
SEM image analysis of cracks in the insulator cement used for 48 years.

**Figure 11:**
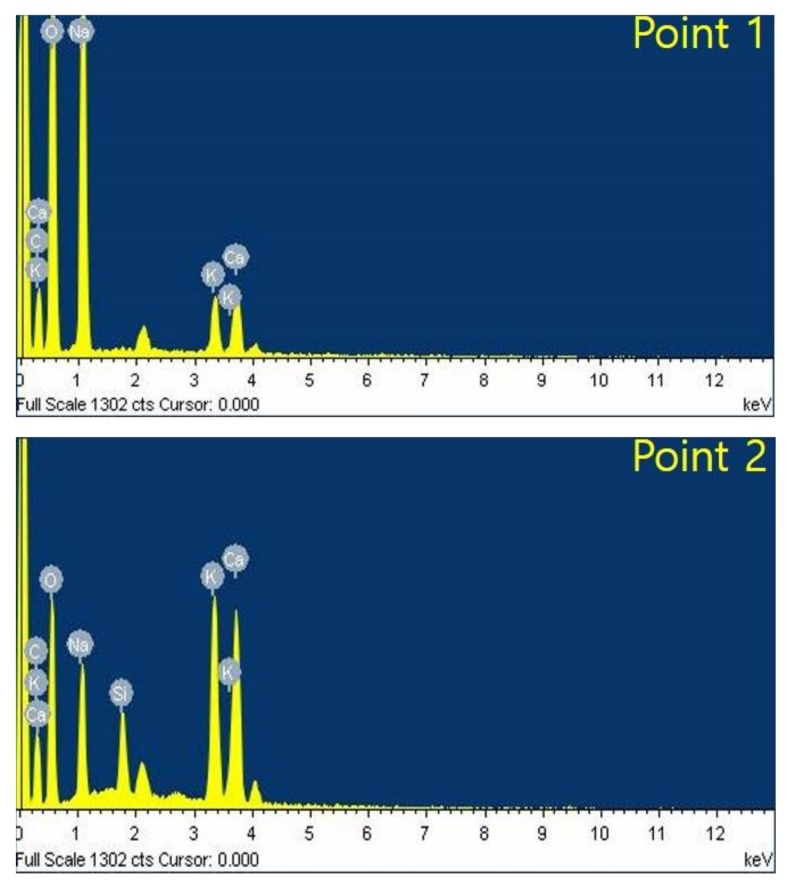
EDS analysis of alkali–silica reaction (ASR) in the insulator cement used for 48 years.

Figure 10 shows the results of the analysis of cracks and expansions caused by ASR in the deteriorated cement of the insulator that was used for 48 years. It can be observed that the cracks in the cement inside the insulator has caused some breakage. Figure 11 EDS analysis of alkali–silica reaction (ASR) in the insulator cement used for 48 years has been depicted.

The content of elemental components is shown in Table 3 for range 1, in which cracking occurs and range 2, in which cracking does not occur.

In the case of crack 1, the ASR decreases and Si, K, and Ca decrease by 2.71%, 5.99%, and 6.19%, respectively. Reactive component Na element increases by 9.88%. It can be observed that Na_2_SiO_3_xH_2_O was formed due to external moisture penetration. The corrosion of cement implies that the mechanical properties of cement have considerably deteriorated. In severe cases, corrosion of the internal pins starts, which poses the risk of mechanical desorption of the porcelain insulator, when the tower is being serviced [49,50].

## 4. Conclusions

The suspension insulator used in 154 kV transmission line comprises iron, porcelain, and cement.This study focused on the deterioration of cement of porcelain insulator, due to ASR and detailed study of ASR occurs in the pores of the cement. Types of defects in cement range from capillary pores of several µm in size to large pores ranging from tens to hundreds of millimeters visible to the naked eye. Cement initially expands and contracts, but when moisture infiltrates, it can be observed that it expands over time, due to ASR. If the internal cracks of the cement are hard to view, fuchsine solution can be easily used to detect these invisible cracks. The degradation of the cement can be observed by spraying a universal indicator to show color variations that correspond to the pH value of the cement. The pH value of cement is initially 12–13, which indicates strong alkalinity; however, when it is used for a long time, and water penetration causes expansion, due to ASR, the pH value indicates a neutral and even an acidic tendency. In porcelain insulators, the cement becomes neutral after 38 years of use and should not be used, because the mechanical strength of these insulators cannot be guaranteed after this time period. After 48 years, the pH value is approximately 7 or a little bit lower, which indicates an acidic tendency. From the scanning electron microscopy-energy dispersive spectroscopy analysis of the cracked part of deteriorated cement, it was observed that ASR showed a 2.71% decrease of Si in total content and 5.99% decrease in K. It showed a 6.19% decrease in Ca and 9.88% increase in reactive Na element. We analyzed the deterioration of cement inside the porcelain insulator. To prevent the occurrence of deterioration, it is also possible to use low alkali cement or form a coating film so that moisture does not penetrate the cement that is exposed to the pin part of the porcelain insulator.

## Figures and Tables

**Table 1:** Dependence of metal corrosion on pH value inside the porcelain insulator.

**Effect by pH value**	**pH Value**	**Effect**
	Higher than 10	Difficult to corrode metal
	Between 4 and 10	High probability of metal corrosion
	Between 1 and 3	Metal is easily corroded

**Table 2:** Ion and hydroxide ion activities on the pH scale.

Properties	pH	H^+^Activity	OH^−^Activity
Acid	0	1 × 10^−0^	1 × 10^−14^
	1	1 × 10^−1^	1 × 10^−13^
	2	1 × 10^−2^	1 × 10^−12^
	3	1 × 10^−3^	1 × 10^−11^
	4	1 × 10^−4^	1 × 10^−10^
Neutral	5	1 × 10^−5^	1 × 10^−9^
	6	1 × 10^−6^	1 × 10^−8^
	7	1 × 10^−7^	1 × 10^−7^
Base	8	1 × 10^−8^	1 × 10^−6^
	9	1 × 10^−9^	1 × 10^−5^
	10	1 × 10^−10^	1 × 10^−4^
	11	1 × 10^−11^	1 × 10^−3^
	12	1 × 10^−12^	1 × 10^−2^
	13	1 × 10^−13^	1 × 10^−1^
	14	1 × 10^−14^	1 × 10^−0^

**Table 3:** Hydrogen ion and hydroxide ion activities on the pH scale.

	C	O	Na	Si	K	Ca
1	19.52	59.96	17.02	-	1.5	2
2	19.88	54.59	7.14	2.71	7.49	8.19
